# Characterization of a major QTL for manganese accumulation in rice grain

**DOI:** 10.1038/s41598-017-18090-7

**Published:** 2017-12-18

**Authors:** Chaolei Liu, Guang Chen, Yuanyuan Li, Youlin Peng, Anpeng Zhang, Kai Hong, Hongzhen Jiang, Banpu Ruan, Bin Zhang, Shenglong Yang, Zhenyu Gao, Qian Qian

**Affiliations:** State Key Laboratory of Rice Biology, China National Rice Research Institute, Hangzhou, 310006 China

## Abstract

Some diets lack sufficient manganese (Mn), an essential mineral. Increasing Mn in grain by biofortification could prevent Mn deficiency, but may increase levels of the toxic element cadmium (Cd). Here, we investigated Mn in rice (*Oryza sativa*) grains in recombinant inbred lines (RILs) from the cross of 93–11 (low grain Mn) with PA64s (high grain Mn). Quantitative trait locus (QTL) analysis to identify loci controlling grain Mn identified a major QTL, *qGMN7.1*, on the short arm of chromosome 7; *qGMN7.1* explained 15.6% and 22.8% of the phenotypic variation in the RIL populations grown in two distinct environments. We validated the QTL with a chromosome segment substitution line (CSSL), CSSL*-qGMN7.1*, in the 93–11 background harboring *qGMN7.1* from PA64s. Compared to 93–11, CSSL*-qGMN7.1* grain had increased Mn and decreased Cd concentrations; CSSL*-qGMN7.1* roots also showed enhanced Mn uptake. Fine mapping delimited *qGMN7.1* to a 49.3-kb region containing *OsNRAMP5*, a gene responsible for Mn and Cd uptake. Sequence variations in the *OsNRAMP5* promoter caused changes in its transcript level, and in grain Mn levels. Our study thus cloned a major QTL for grain Mn concentration in rice, and identified materials for breeding rice for high Mn and low Cd concentrations in the grain.

## Introduction

Manganese (Mn) is an essential trace element for plants, domestic animals, and humans^1^. In mammals, Mn is necessary for proper fetal development and growth and is crucial throughout the life span^2,3^. Humans maintain stable tissue levels of Mn and Mn is present in virtually all diets at low concentrations^4^. The U.S. National Research Council has established an estimated safe and adequate dietary Mn intake of 2–5 mg per day for adults^5^. Overt Mn deficiency diseases are extremely rare, but segments of populations from remote areas still suffer from subclinical Mn deficiency^1,6^. Cadmium (Cd) is a toxic heavy metal that is harmful to human health and Mn^2+^ and Cd^2+^ can be transported by the same transporters^7^. The international maximum limit for rice is 0.4 mg Cd·kg^−1^ polished rice^8^. As rice is the staple food for over half of the world’s population, production of rice with adequate levels of Mn and low levels of Cd would benefit human health.

Similar to other elements, Mn accumulation in rice grains occurs via root uptake, vascular transport, and transfer into the developing grain. The available Mn in the soil rhizosphere is first taken up by transporters in the plasma membrane of root cells, then loaded into the xylem, and transferred to shoots via the xylem. It is distributed to different storage compartments regulated by phloem loading and mobility, and finally remobilized from other organs, such as leaves, to the grains at the filling stage^9^. Many factors can affect these processes and ultimately determine the Mn level in grains, among which, genetics appears to be the dominant factor.

The use of reverse genetic approaches has uncovered several genes involved in Mn transport, translocation, or distribution in rice^10–12^. *OsYSL2* encodes a functional iron [Fe(II)]- and Mn [Mn(II)]-nicotianamine complex transporter expressed in phloem cells and developing seeds, and OsYSL2 is required for long-distance transport of Fe and Mn^10^. OsNRAMP3 and OsNRAMP5 belong to the natural resistance-associated macrophage protein (NRAMP) family^11,12^. OsNRAMP3 is a node-localized Mn transporter that functions as a switch for regulating Mn distribution in rice^11^. OsNRAMP5 regulates Mn uptake and the accumulation of Mn in rice^12^. OsNRAMP5 also functions in iron (Fe) and Cd transport^12–15^. Knockout or mutation of *OsNRAMP5* greatly decreased Cd uptake by the roots, resulting in a decline in the Cd concentration in the straw and grain^12,13^.

In the last decade, quantitative trait locus (QTL) mapping for grain Mn concentration with various mapping populations has identified a number of QTLs in rice^16–24^. Several major QTLs on chromosomes 3, 7, and 8 have been detected. For example, the major QTL, *qMNCN-3*, on chromosome 3, identified by Shen^17^, was also detected as *qMn3* by Du *et al*.^22^, explaining 9% and 10.8% of the phenotypic variation in the two studies, respectively. *qMNCN-8*, on the short arm of chromosome 8, was also detected by different studies^17,22,23^. However, to date, no QTL associated with grain Mn concentration has been fine mapped or cloned.

The hybrid cultivar Liang-You-Pei-Jiu (LYPJ) was produced by a cross between the paternal parent 93–11 (an *indica* variety widely grown in China), and the maternal parent PA64s (with a mixed genetic background of *indica* and *japonica*). This pioneer super hybrid yielded 10.5 tons/ha in 2000^25^. We previously constructed 132 LYPJ-derived core recombinant inbred lines (RILs) and re-sequenced them to establish a high-density single-nucleotide polymorphism (SNP) linkage map^26^. Here, we performed a QTL analysis for grain Mn concentration based on the previously developed genetic map and characterized a major QTL controlling Mn accumulation in rice grains.

## Results

### The *qGMN7.1* from PA64s significantly increased grain Mn concentration

The RIL population from the rice super hybrid LYPJ, and the hybrid parents were grown in two different environments, Hainan (110.0 E, 18.5 N) and Hangzhou (120.0 E, 30.1 N), China. Mature seeds were harvested for determining the grain Mn concentration. The concentration of Mn in the grains was significantly different between the parents in both Hainan and Hangzhou, with concentrations in PA64s approximately 2-fold higher than in 93–11 (Fig. S1 and Table S1). The RIL population showed a wide range of phenotypic variation, in a continuous distribution (Fig. S1). Using the high-resolution SNP map, we detected 12 QTLs for grain Mn concentration distributed on all chromosomes except for chromosomes 10, 11, and 12 (Fig. S2 and Table S2). Among those QTLs, 5 were identified in the RIL populations grown in both Hainan and Hangzhou, and 8 had additive effects coming from PA64s. One major QTL with the highest LOD value, *qGMN7.1*, was mapped between markers SNP7-53 and SNP7-64 on the short arm of chromosome 7 and explained 15.6% and 22.8% of the phenotypic variation in the RIL populations grown in Hangzhou and Hainan, respectively (Fig. S2 and Table S2).

**Figure 1:**
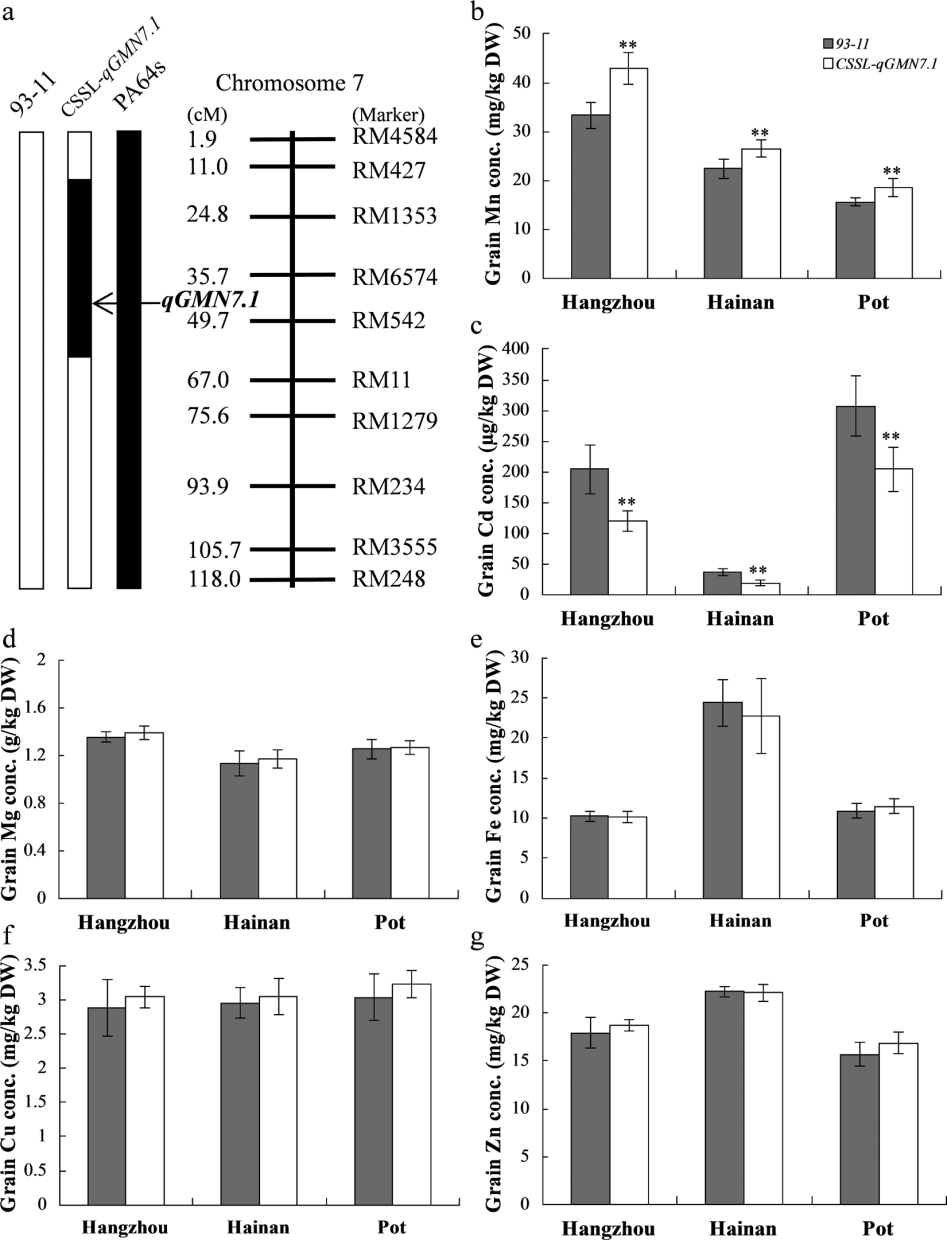
*qGMN7.1* from PA64s significantly increased grain Mn concentration. (**a**) Schematic graph of chromosome 7 of CSSL*-qGMN7.1* and the two parents. The white and black bars represent 93–11 and PA64s alleles, respectively. The name and genetic locus of the SSR markers and the position of *qGMN7.1* are indicated. (**b**–**g**) Concentrations of Mn, Cd, Mg, Fe, Cu, and Zn in the grains of 93–11 (grey) and CSSL*-qGMN7.1* (white) grown in the field of Hangzhou and Hainan, China, and in pots in Hangzhou (2015). Vertical bars represent the standard deviation (*n* = 6). **Indicates a 1% significance level according to the *t* test.

To confirm the effect of *qGMN7.1* on grain Mn concentrations, we constructed a chromosome segment substitution line (CSSL), CSSL-*qGMN7.1*, in the 93–11 background (Fig. S3), harboring the segment from PA64s between the markers RM427 and RM11 (Fig. 1a). When 93–11 and CSSL-*qGMN7.1* were cultivated in the paddy fields in Hangzhou and Hainan or grown in pots in Hangzhou (2015), the concentration of Mn in the CSSL-*qGMN7.1* grain was significantly higher than that of 93–11 (Fig. 1b). Interestingly, CSSL-*qGMN7.1* also showed decreased Cd concentrations in the grain, compared to 93–11 (Fig. 1c). The concentrations of other trace metals in the grains, such as magnesium (Mg), Fe, zinc (Zn), and copper (Cu), were not significantly different between CSSL-*qGMN7.1* and 93–11 (Fig. 1d–g).

### Physiological characteristics of CSSL*-qGMN7.1*

**Figure 2:**
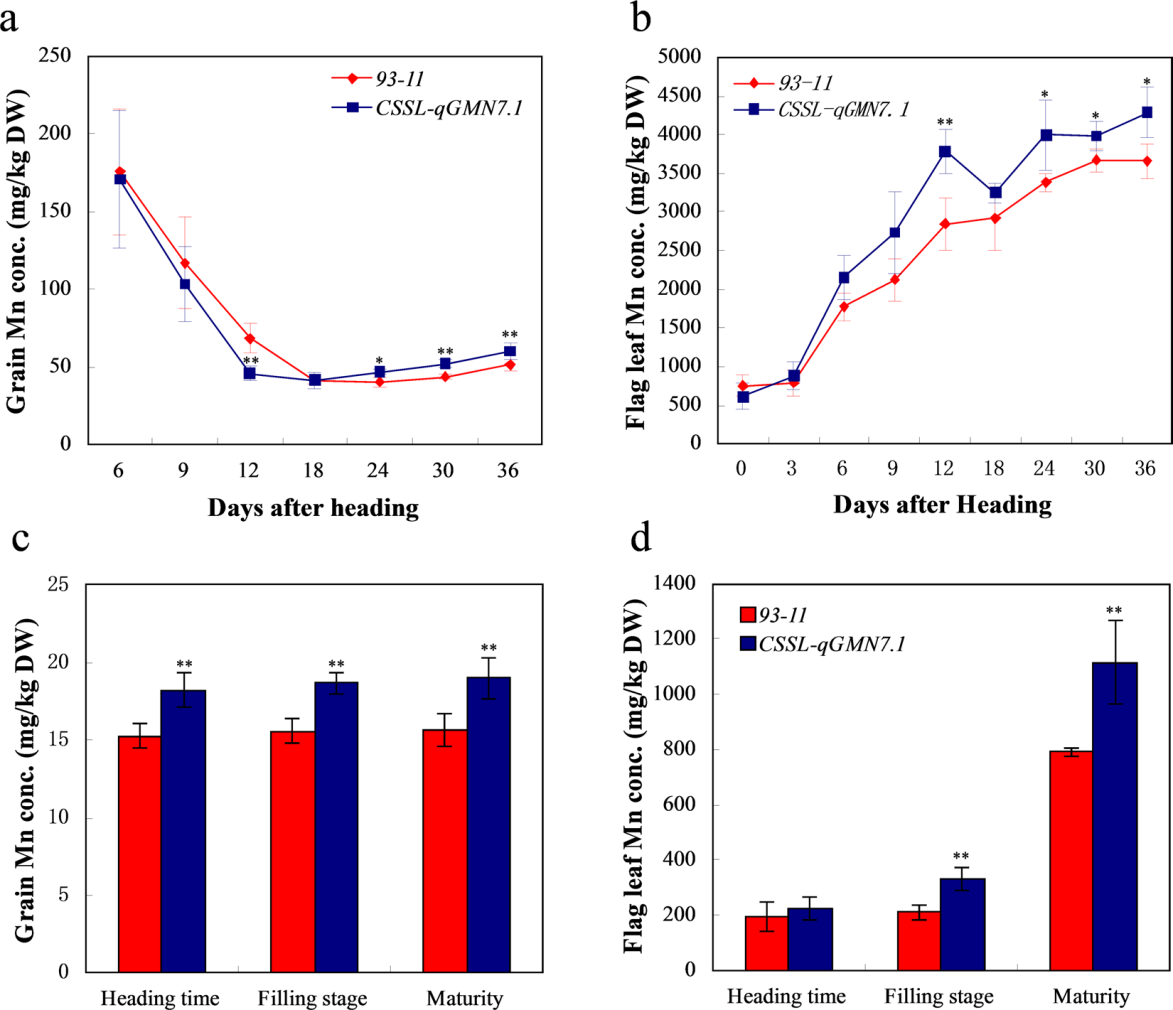
Comparison of time-course experiment of Mn accumulation in the grain and flag leaf between 93–11 and CSSL*-qGMN7.1*. (**a**,**c**) Grain Mn concentration of 93–11 (red) and CSSL*-qGMN7.1* (blue). Grains were harvested at 6, 9, 12, 18, 24, 30, and 36 days after heading (**a**), grains were tagged and harvested until maturity after their corresponding flag leaves were sampled at the heading, grain-filling, and maturity stages (**c**). (**b**,**d**) Flag leaf Mn concentration of 93–11 (red) and CSSL*-qGMN7.1* (blue). Flag leaves were harvested at 0, 3, 6, 9, 12, 18, 24, 30, and 36 days after heading (**b**), flag leaves were sampled at the heading, grain-filling, and maturity stages (**d**). Vertical bars represent the standard deviation (*n* = 4). *and **indicate a 5% and 1% significance level, respectively, according to the *t* test.

We performed a series of physiological experiments to determine the physiological mechanism underlying the increased grain Mn concentration conferred by *qGMN7.1*. In a time-course experiment of grain Mn accumulation, no significant difference was found between 93–11 and CSSL-*qGMN7.1* at the early grain-filling stage, although both lines showed decreasing accumulation with time (Fig. 2a). At the 18th day after heading, the grain Mn concentration stabilized and then significantly increased from the 24th day after heading in CSSL*-qGMN7.1* compared to 93–11. The difference remained significant through maturity (Fig. 2a). Overall, CSSL*-qGMN7.1* and 93–11 had a similar pattern of Mn accumulation, and *qGMN7.1* functioned during the late grain-filling stage.

We also measured Mn concentrations in flag leaves at different times after heading and found that Mn increased in both CSSL*-qGMN7.1* and 93–11, with higher levels in CSSL*-qGMN7.1*, from the late filling stage to maturity (Fig. 2b). To determine whether Mn is transferred from other organs, such as the flag leaf, into the grain at the late filling stage, we removed flag leaves and measured the effect on Mn in the grains. Removal of flag leaves at the heading and filling stages did not affect Mn accumulation in the grains (Fig. 2c, d). These results suggested that Mn is not transferred from flag leaves to the grains at the grain-filling through maturity stages.

**Figure 3:**
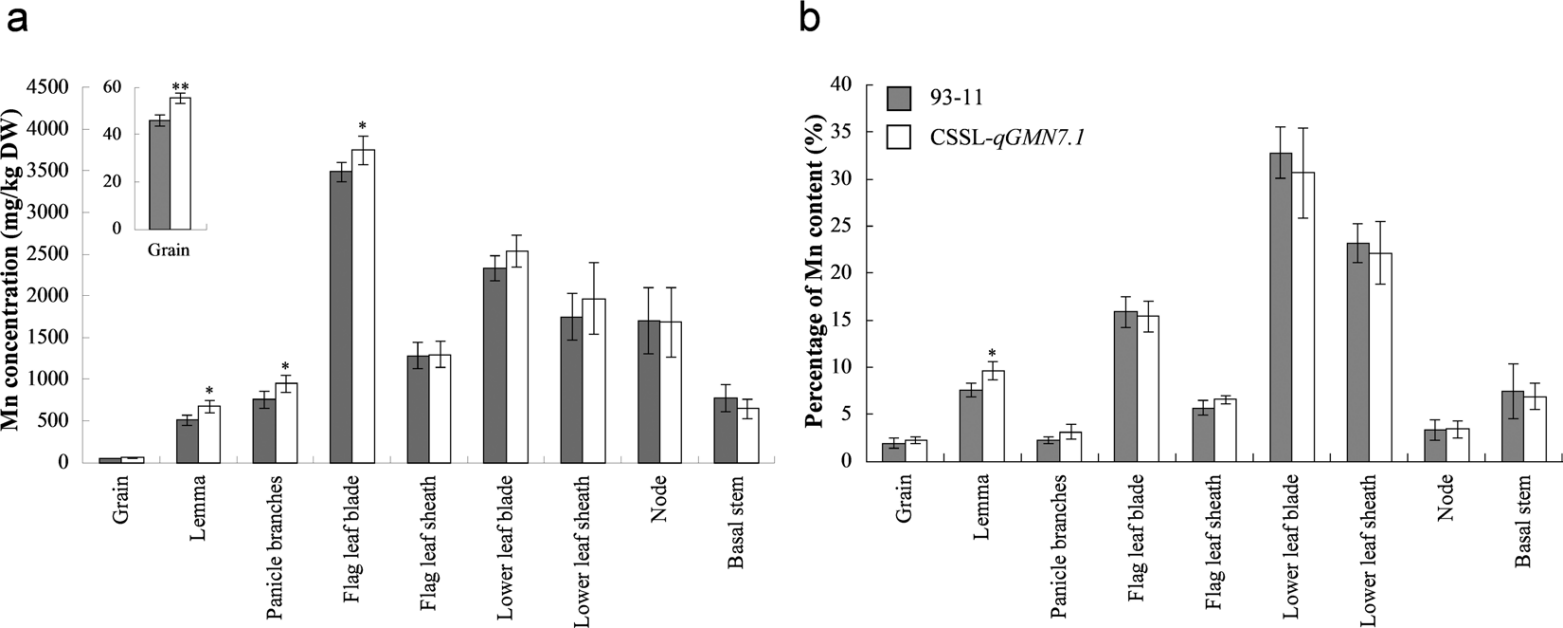
Mn distribution in the aboveground tissues of 93–11 and CSSL*-qGMN7*. (**a**) Mn concentration in different organs. (**b**) Proportion of Mn content in different organs to the Mn content in the whole plant (except roots). The aboveground tissues of 93–11 (grey) and CSSL*-qGMN7.1* (white) grown in the paddy field were harvested and separated into different organs at maturity. Vertical bars represent the standard deviation (*n* = 4). *and **indicate a 5% and 1% significance level, respectively, according to the *t* test.

As the distribution of Mn in the aboveground parts has been reported to play an important role in grain Mn accumulation, we analyzed the Mn concentration in different organs at maturity. The highest Mn concentration was observed in flag leaf blades, with approximately 3,500 mg·kg^−1^ dry weight (DW), and the lowest in the grains with only about 50 mg·kg^−1^ DW in the rice plants (Fig. 3a). Compared to 93–11, CSSL*-qGMN7.1* accumulated higher concentrations of Mn in the grains, lemma, panicle branches, and flag leaf blades. However, Mn concentrations in other leaves and stems were almost the same in both lines (Fig. 3a). An analysis of the proportion of Mn content in different organs to the whole plant showed that the proportion of grain Mn content/whole plant Mn content was similar between CSSL*-qGMN7.1* and 93–11 (Fig. 3b).

**Figure 4:**
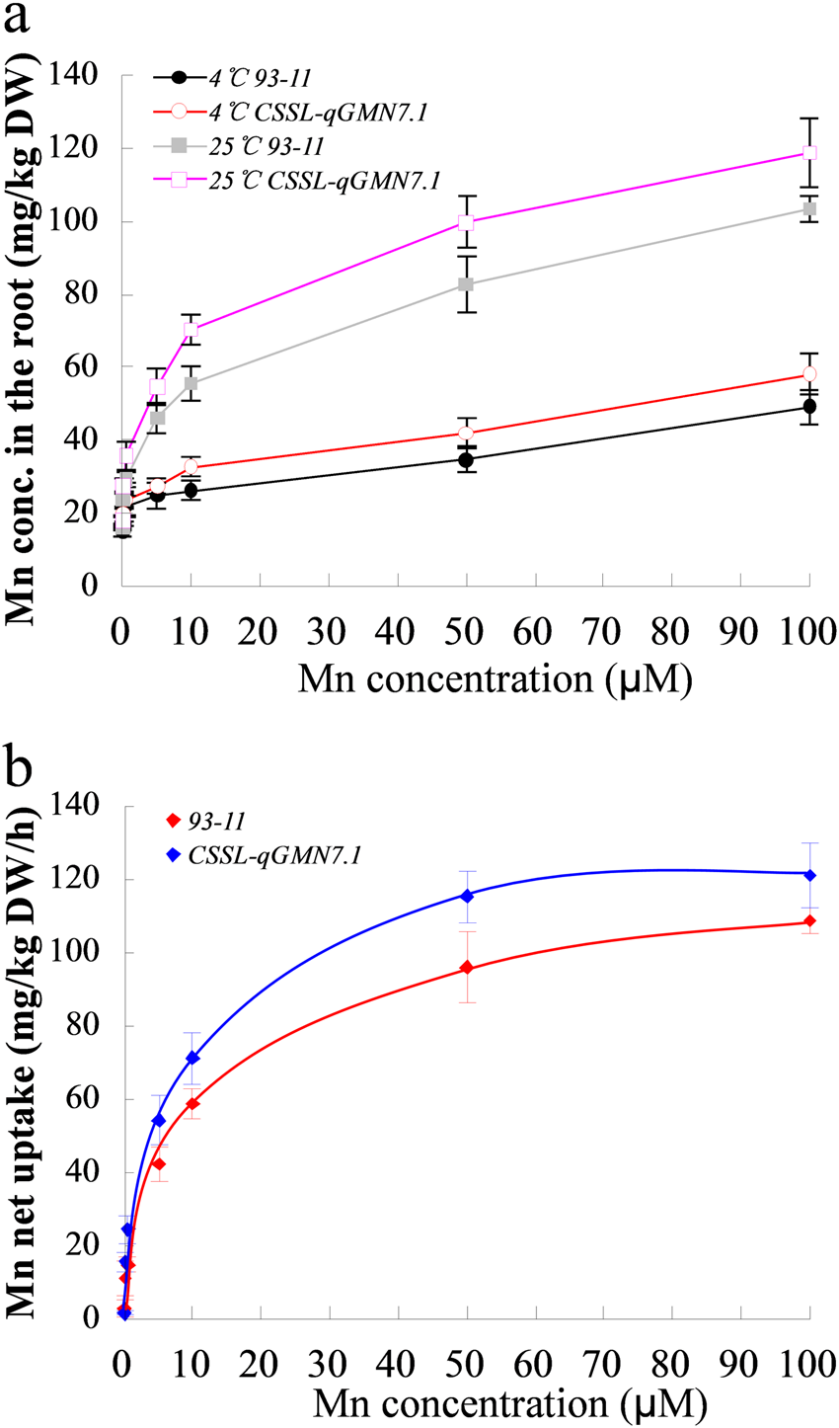
Short-term uptake of Mn by rice roots. (**a**) Prepared seedlings of 93–11 (closed symbols) and CSSL*-qGMN7.1* (open symbols) were exposed to nutrient solutions containing various concentrations of Mn (0, 0.1, 0.5, 5, 10, 50, and 100 µM) for 30 min at 25 °C (square) or 4 °C (circle). (**b**) Net uptake of Mn was calculated by subtracting the uptake at 4 °C from that at 25 °C. Red rhombus, 93–11; blue rhombus, CSSL*-qGMN7.1*. Red and blue lines represent Michaelis-Menten curves for 93–11 and CSSL*-qGMN7.1*, respectively. Error bars represent standard deviation (*n* = 4).

These results indicate that the higher Mn concentration in the grains of CSSL*-qGMN7.1* did not result from greater distribution from other organs, but from higher assimilation in the roots. To confirm this, we performed a short-term (30 min) uptake experiment using intact roots of 93–11 and CSSL*-qGMN7.1*. The Mn uptake at 4 °C was much lower than that at 25 °C in both lines, but CSSL*-qGMN7.1* exhibited higher Mn uptake than 93–11 at both temperatures (Fig. 4a). The net Mn uptake, calculated by subtracting the Mn uptake at 4 °C from that at 25 °C, was significantly greater in CSSL*-qGMN7.1* than in 93–11 (Fig. 4b). Although CSSL*-qGMN7.1* and 93–11 had a similar affinity for Mn, the value of V_max_ for CSSL*-qGMN7.1* (118.6 mg·kg^−1^ root DW·h^−1^) was significantly higher than that for 93–11 (103.4 mg·kg^−1^ root DW·h^−1^) (Fig. 4).

### Fine mapping of the *qGMN7.1* to a 49.3-kb genomic region

**Figure 5:**
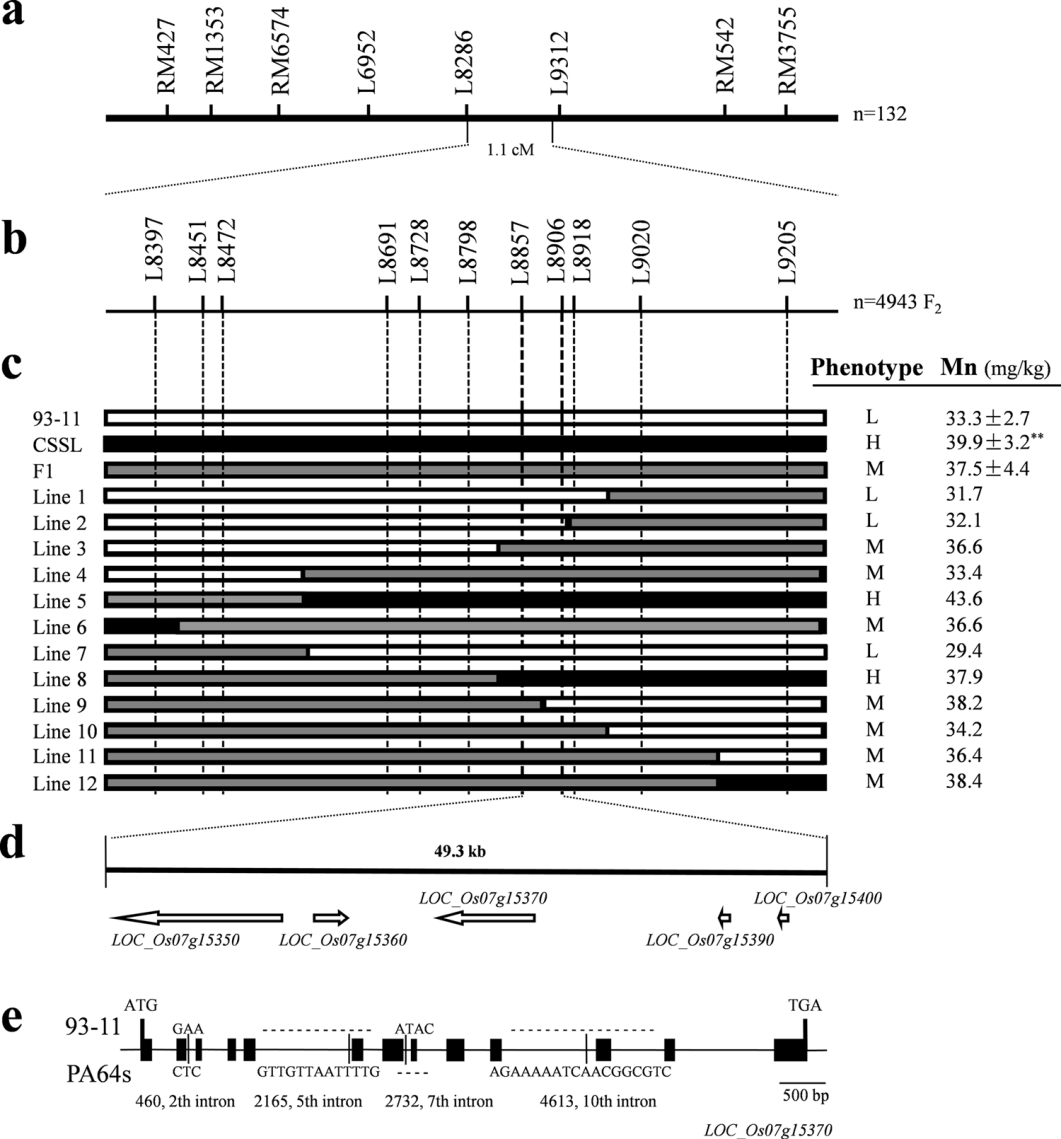
Fine mapping of *qGMN7.1*. (**a**) *qGMN7.1* was mapped between L8286 and L9312 based on LYPJ RILs. (**b**) 4,943 segregating individuals derived from a cross between 93–11 and CSSL*-qGMN7.1* were used for fine mapping. (**c**) Genotyping and phenotyping of recombinants delimited *qGMN7.1* to within a 49.3-kb region flanked by L8857 and L8906. Phenotype for grain Mn concentration: L (low), M (medium), or H (high). (**d**) RiceGAAS predicted five genes in the target region. (**e**) *LOC_Os07g15370* structure and sequence variations between two parents. **Indicates a 1% significance level compared to 93–11 according to the *t* test (*n* = 6).

To fine map *qGMN7.1*, CSSL-*qGMN7.1* was crossed with 93–11 to generate a segregating population. The F_1_ plants of this cross showed intermediate concentrations of grain Mn, indicating semi-dominant inheritance of *qGMN7.1* (Fig. 5c). We planted 4,943 individuals in Hainan (2015) and screened them with markers L8286 and L9312 flanking *qGMN7.1* and 12 recombinants were identified (Fig. 5). Nine newly developed insertion/deletion (InDel) markers, well distributed within the interval, were used to further genotype the 12 recombinants (Fig. 5b and Table S3). We tested the F_2:3_ progeny of the recombinants in Hangzhou in 2016. Grain Mn concentrations showed no significant difference among the three genotypes of recombinants Line 1, Line 2, and Line 8 (Table 1). For the other recombinant lines, *qGMN7.1* segregated and significant differences in phenotypes were found in the three genotypes of *qGMN7.1* (Table 1). Based on the phenotypes and genotypes of these recombinants, we delimited *qGMN7.1* to a region of approximately 49.3-kb between markers L8857 and L8906 (Fig. 5c, d).

**Table 1:** Progeny test of nine recombinants for confirmation of the fine-mapped region of *qGMN7.1*.

Recombinants		Markers								Grain Mn conc. (mg/kg DW)	Grain Cd conc. (µg/kg DW)
		L8397	L8472	L8798	L8857	L8906	L8918	L9020	L9205		
Line 1	Type 1	9	9	9	9	9	9	P	P	41.36 ± 2.72	507.34 ± 50.55
	Type 2	9	9	9	9	9	9	H	H	40.65 ± 4.03	467.32 ± 59.49
	Type 3	9	9	9	9	9	9	9	9	42.00 ± 4.10	506.39 ± 68.26
Line 2	Type 1	9	9	9	9	9	P	P	P	42.30 ± 3.94	481.06 ± 72.41
	Type 2	9	9	9	9	9	H	H	H	38.60 ± 3.73	462.25 ± 85.67
	Type 3	9	9	9	9	9	9	9	9	40.89 ± 3.91	464.53 ± 83.16
Line 3	Type 1	9	9	9	9	P	P	P	P	55.58 ± 4.37	578.19 ± 61.15
	Type 2	9	9	9	9	H	H	H	H	46.24 ± 3.86**	620.75 ± 69.83
	Type 3	9	9	9	9	9	9	9	9	46.87 ± 3.37**	552.72 ± 44.53
Line 4	Type 1	9	9	P	P	P	P	P	P	48.47 ± 3.99	508.47 ± 61.99
	Type 2	9	9	H	H	H	H	H	H	41.30 ± 4.74	488.22 ± 66.23
	Type 3	9	9	9	9	9	9	9	9	38.85 ± 4.32**	513.40 ± 55.27
Line 6	Type 1	P	9	9	9	9	9	9	9	46.38 ± 4.02	273.19 ± 53.55
	Type 2	P	H	H	H	H	H	H	H	52.80 ± 5.70	310.51 ± 26.32
	Type 3	P	P	P	P	P	P	P	P	58.85 ± 5.14**	284.98 ± 68.59
Line 8	Type 1	9	9	9	P	P	P	P	P	53.86 ± 4.83	528.79 ± 63.56
	Type 2	H	H	H	P	P	P	P	P	56.57 ± 3.92	368.06 ± 99.61**
	Type 3	P	P	P	P	P	P	P	P	56.99 ± 5.19	407.64 ± 49.28**
Line 9	Type 1	P	P	P	P	9	9	9	9	51.05 ± 4.14	341.01 ± 68.95
	Type 2	H	H	H	H	9	9	9	9	46.75 ± 4.71	387.54 ± 62.21
	Type 3	9	9	9	9	9	9	9	9	43.57 ± 3.64*	499.33 ± 64.07**
Line 10	Type 1	P	P	P	P	P	9	9	9	49.71 ± 5.74	389.76 ± 86.87
	Type 2	H	H	H	H	H	9	9	9	42.39 ± 3.73*	428.91 ± 57.30
	Type 3	9	9	9	9	9	9	9	9	41.03 ± 2.94**	599.43 ± 69.62**
Line 12	Type 1	9	9	9	9	9	9	9	P	42.77 ± 3.89	648.47 ± 91.99
	Type 2	H	H	H	H	H	H	H	P	46.87 ± 5.39	488.22 ± 76.23**
	Type 3	P	P	P	P	P	P	P	P	50.89 ± 5.29*	413.40 ± 95.27**

Note: Type 1, type 2, and type 3 in each panel represent the segregated genotypes of the recombinants. ‘9’, ‘P’, and ‘H’ represent the homozygote of 93–11 and PA64s, and the heterozygote of the parents, respectively. *and **indicate a 5% and 1% significance level compared to type I, respectively, according to the *t* test (*n* = 6).

We also measured the Cd concentration in the grains of the F_2:3_ progeny of the recombinants. Three genotypes in the recombinants Line 1 to Line 4 and Line 6 showed no significant difference in grain Cd concentration, and recombinants Line 8 to Line 10 and Line 12 exhibited segregating phenotypes (Table 1). Combined with the genetic recombination sites of these recombinants (Fig. 5c), we concluded that *qGMN7.1* had little influence on Cd accumulation in the grain. Furthermore, the Cd concentration in the grain of recombinant Line 6 was about 300 µg·kg^−1^ DW, much lower than that of recombinant Line 1 to Line 4 (500 to 600 µg·kg^−1^ DW), indicating that the allele from PA64s could greatly decrease grain Cd concentration.

### *OsNRAMP5* is the candidate gene for the grain Mn accumulation trait

The Rice Genome Annotation Project (http://rice.plantbiology.msu.edu/) predicted five genes in the 49.3-kb target region of *qGMN7.1* (Fig. 5d): *LOC_Os07g15350* encoding a transposon, *LOC_Os07g15360* and *LOC_Os07g15390* encoding retrotransposons, *LOC_Os07g15400* encoding an expressed protein, and *LOC_Os07g15370* encoding a metal transporter. Because *LOC_Os07g15370* has been reported previously as *OsNRAMP5*, encoding a major transporter for Mn and Cd^12^, it was considered the most likely candidate gene for the grain Mn accumulation trait in *qGMN7.1*.

**Figure 6:**
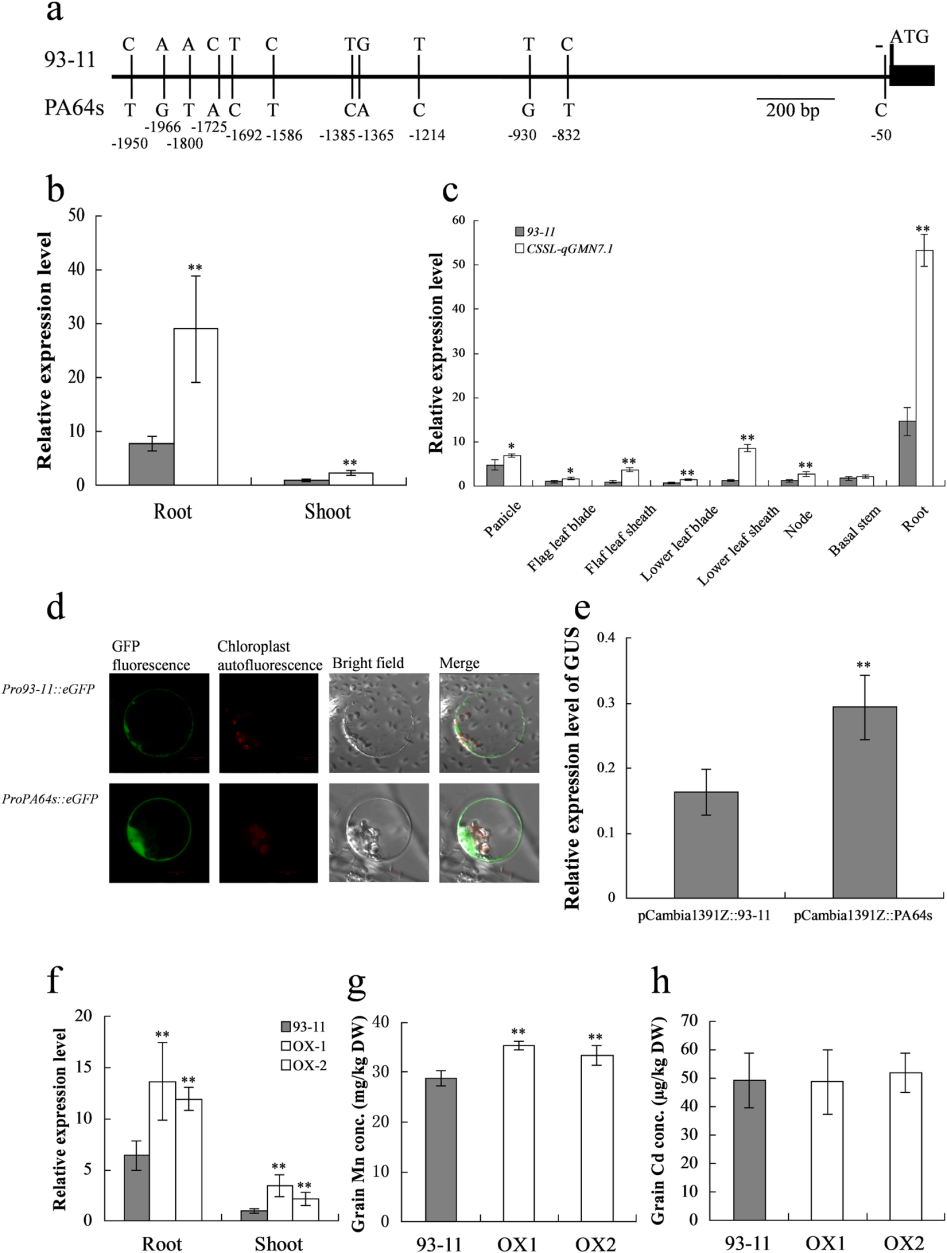
Validation of candidate gene. (**a**) Sequence variations in the promoter region of *OsNRAMP5* between two parents. (**b**,**c**) Comparison of the relative expression level of *OsNRAMP5* between 93–11 (grey) and CSSL-*qGMN7.1* (white) in roots and shoots at the seedling stage (**b**), and in different organs at the booting stage (**c**). (**d**,**e**) Promoter activity analysis. The green fluorescent signals of GFP driven by the *OsNRAMP5* promoter from 93–11 and PA64s (**d**), the relative expression level of *GUS* driven by the *OsNRAMP5* promoters from 93–11 and PA64s (**e**). (**f**–**h**) Relative expression level of OsNRAMP5 and grain Mn and Cd concentrations in 93–11 and the overexpression (OX) lines grown in pots. *and **indicate a 5% and 1% significance level, respectively, according to the *t* test.

Sequence alignment of *OsNRAMP5* between the two parents, 93–11 and PA64s, revealed no polymorphisms in the coding sequence, but 12 variations in the promoter region (Figs 5e and 6a). These sequence variations might alter the transcript levels and be responsible for the different phenotypes. Therefore, we measured the expression levels of *OsNRAMP5* in 93–11 and CSSL-*qGMN7.1* at different developmental stages. At the seedling stage, CSSL-*qGMN7.1* had significantly higher transcript levels of *OsNRAMP5* than in 93–11, with the largest difference of 3.7-fold found in the roots. Higher transcript levels were also observed in CSSL-*qGMN7.1* at the booting stage, particularly in the roots (Fig. 6b, c). We then compared the promoter activity of *OsNRAMP5* between 93–11 and PA64s by transient expression in rice protoplasts. The green fluorescent signals of GFP driven by the PA64s promoter were stronger than those driven by the 93–11 promoter (Figs 6d and S4), and the *GUS* transcript levels driven by the PA64s promoter also showed a higher level compared to that driven by the 93–11 promoter (Fig. 6e). These results suggested that the *OsNRAMP5* promoter from PA64s was stronger than the *OsNRAMP5* promoter from 93–11.

To validate that *OsNRAMP5* is responsible for Mn accumulation in the grain, we overexpressed it in 93–11. A significantly larger abundance of *OsNRAMP5* transcript was found in roots (2 fold) and shoots (2–3 fold) of the overexpression lines than in 93–11 (Fig. 6f). The overexpression lines accumulated more Mn in the grains than 93–11 when grown in pots (Fig. 6g). However, the Cd concentration in the grains was nearly equal in 93–11 and the overexpression lines (Fig. 6h). Therefore, we concluded that *OsNRAMP5* is responsible for the grain Mn accumulation trait in *qGMN7.1*.

**Figure 7:**
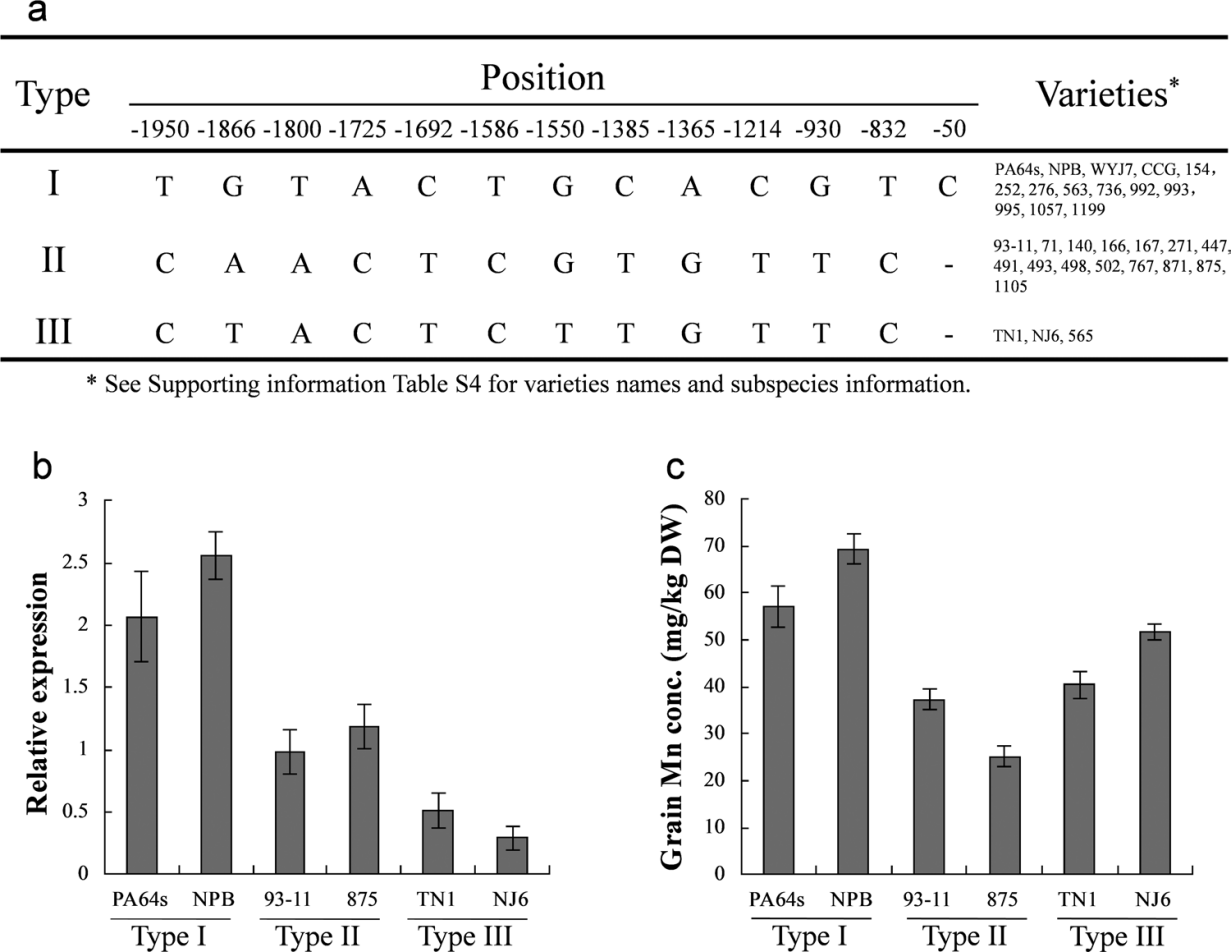
Haplotype analysis of the *OsNRAMP5* promoter in different rice varieties. (**a**) *OsNRAMP5* promoter haplotypes of rice varieties used in the study. (**b**) Relative expression level of *OsNRAMP5* in the varieties for 3 haplotypes, the expression level was detected in the flag leaves at the grain-filling stage. (**c**) Grain Mn concentration in the varieties for 3 haplotypes.

To gain further insight into the variations of the *OsNRAMP5* promoters, we isolated and compared the 2-kb 5′-flanking regions of *OsNRAMP5* from 30 different rice varieties. Based on the promoter sequences, three haplotypes were identified (Fig. 7a and Table S4). Among the 30 rice varieties, 13 had the same haplotype as PA64s (designated as type I) and 14 coincided with that of 93–11 (designated as type II). The promoters from varieties TN1, NJ6, and No.565 were consistent with type II, with the exception of nucleotide variations at positions −1,866 (A → T) and −1,550 (G → T) from the start codon ATG (these were designated as type III) (Fig. 7a). Compared to type I, the rice varieties containing type II and III promoters exhibited lower expression of *OsNRAMP5* and lower accumulation of Mn in the grains (Fig. 7b, c).

## Discussion

To date, hundreds of QTLs related to grain mineral elements have been identified in rice, but few have been fine mapped or cloned. In this study, we analyzed the Mn concentration in the grains of the RILs derived from the rice super hybrid LYPJ and found 12 putative QTLs in two testing environments. Among them, *qGMN7.1*, detected on the short arm of chromosome 7 in both environments, accounted for the largest proportion of phenotypic variation (Fig. S2 and Table S2). In the same chromosomal region, a major QTL was also detected by Ishikawa as *qGMn7* using rice varieties Sasanishiki and Habataki as parents^20^, suggesting *qGMN7.1* might be a common genetic factor for grain Mn concentrations in various rice varieties.

Previous studies showed that Mn content was significantly correlated with the contents of other mineral elements in grains, such as Mg, Fe, Zn, or Cu, suggesting that the contents of these elements might be controlled by common genes^23,24^. However, the concentrations of Mg, Fe, Zn, and Cu in the grains were nearly equal between CSSL-*qGMN7.1* and 93–11 (Fig. 1d–g). Though the Cd concentration in the grains of CSSL-*qGMN7.1* was much lower than that of 93–11, fine mapping of *qGMN7.1* demonstrated that it had little influence on Cd accumulation in the grains (Table 1). These results implied that *qGMN7.1* might be specialized for controlling Mn accumulation in the grains. Considering that agronomic traits could also affect the accumulation of elements in grains^27,28^, we investigated 9 traits of 93–11 and CSSL-*qGMN7.1* and found no significant differences between them (Table S5). Therefore, CSSL*-qGMN7.1* is an ideal material for rice breeding due to its improved Mn concentration and decreased Cd concentration in the grains without an accompanying loss of yield.

The role of *OsNRAMP5* in controlling Mn uptake and transport has been reported in rice^12–15,29^. *OsNRAMP5* was constitutively expressed in the roots and encodes a plasma membrane-localized protein that belongs to the natural resistance associated macrophage protein (NRAMP) family, whose members function as proton-coupled metal ion transporters that can transport Mn^2+^, Zn^2+^, Cu^2+^, Fe^2+^, Cd^2+^, Ni^2+^, Co^2+^, and Al^3+^ ^12,30^. *OsNRAMP5* encodes a major transporter responsible for Mn uptake in rice; knockout of *OsNRAMP5* resulted in a significant decline in grain Mn concentrations compared with the wild type^12^. Ishimaru *et al*. also suggested that *OsNRAMP5* could play a role in Mn transport during flowering and seed development^15^.

Based on the RILs and the backcross population, we fine mapped *qGMN7.1* to a 49.3-kb region containing *OsNRAMP5* (Fig. 5d and Table 1). Although we did not find any alterations in the coding sequence of *OsNRAMP5* between 93–11 and PA64s, we did find nucleotide differences in the promoter region (Figs 5e and 6a). These sequence variations lead to differences in the expression level of *OsNRAMP5* and in Mn accumulation in the grains between CSSL*-qGMN7.1* and 93–11 (Fig. 6b, c). Overexpression of *OsNRAMP5* in the 93–11 variety increased the grain Mn concentration (Fig. 6f, g). Therefore, we inferred that the expression level of *OsNRAMP5* contributed to Mn accumulation in the grains. Variations in promoter sequences commonly lead to phenotypic variation in rice^31–35^. The expression of *OsNRAMP1* in roots was higher in high Cd-accumulating varieties (Habataki, Anjana Dhan, Jarjan) compared to low Cd-accumulating varieties (Sasanishiki, Nipponbare) due to a 400-bp deletion in the promoter region of *OsNRAMP1* in the high Cd-accumulating varieties^31^. Consistent with previous reports, we also found that some low Mn-accumulating varieties had similar *OsNRAMP5* promoter sequences as 93–11, which contains low concentrations of Mn in the grains, whereas high Mn-accumulating varieties, including Nipponbare, exhibited promoter sequences similar to PA64s, which is known for high grain Mn concentrations (Fig. 7).

Four major transport processes are involved in the accumulation of mineral elements: (1) root uptake, (2) root-to-shoot translocation by xylem flow, (3) distribution in aboveground tissues, and (4) remobilization from leaves^9^. In our study, Mn content in the flag leaves increased from the heading stage to maturity in the time-course experiment (Fig. 2b). Removal of the flag leaves at the heading and grain-filling stages did not affect Mn accumulation in the grains (Fig. 2c, d). Therefore, we speculated that elevation of Mn concentrations in the grain did not occur due to remobilization from the leaves. In addition, CSSL-*qGMN7.1* and 93–11 showed little difference in Mn distribution (Fig. 3). However, higher Mn uptake activity was found in CSSL-*qGMN7.1* compared to 93–11 (Fig. 4). Considering that *OsNRAMP5* was constitutively expressed in roots (Fig. 6b, c) and that its expression was higher in CSSL-*qGMN7.1* compared with 93–11, we concluded that *OsNRAMP5* is responsible for the increased Mn concentrations in the grain by enhancing Mn uptake in roots.

OsNRAMP5 has also been reported to function as a Cd/Fe transporter^12–14,36,37^. *OsNRAMP5*-knockdown rice lines accumulated more Cd in the shoots, but the total Cd content was lower than in the wild-type plants^36^. The *OsNRAMP5* knockout line lost the ability to take up Mn and Cd concurrently^12^, and the *osnramp5* mutant exhibited decreased Cd (as well as Mn) concentrations in the straw and grain^13^. These studies demonstrated that the entry of Cd into rice root cells occurred via this Mn transporter, OsNRAMP5. However, in our study, CSSL*-qGMN7.1* accumulated lower amounts of Cd than 93–11, contrary to the higher amounts of Mn in CSSL*-qGMN7.1* compared to 93–11 (Fig. 1b, c). The recombinants that showed segregation at the *qGMN7.1* region exhibited no significant differences in grain Cd concentrations (Table 1), and the overexpression lines of *OsNRAMP5* did not accumulate more Cd in the grains compared with 93–11 (Fig. 6h). A possible explanation is that when plants are grown on relatively high-Mn and low-Cd soils (Hangzhou, pH = 6.04 ± 0.02; 480.35 ± 51.02 mg/kg Mn; 0.64 ± 0.12 mg/kg Cd), Cd does not readily accumulate in the grains. Additionally, an antagonistic effect may exist between Mn and Cd uptake. That is, OsNRAMP5 is mainly responsible for the transport of Mn, but not Cd when Mn is abundant. Alternatively, another locus may exist for grain Cd concentration in the substituted segments of CSSL*-qGMN7.1* (Table 1), which displayed lower Cd accumulation levels when compared with 93–11.

## Materials and Methods

### Plant materials, and field and pot experiments

The 132 RILs of LYPJ were developed by a cross between an elite *Oryza sativa* ssp. *indica* inbred variety, 93–11, with low grain Mn concentration and an *Oryza sativa* ssp*. indica* photo-thermo-sensitive male sterile line, PA64s, with high grain Mn concentrations. The RILs and the two parents were grown in the field of CNRRI in Hangzhou (2013) and Hainan (2013), China, and the RIL population was in the F_11_ and F_12_ generation, respectively. For the determination of grain Mn, mature seeds of each line were collected from 6 plants in the middle row.

The chromosomal segment substitution line, CSSL*-qGMN7.1*, was selected from the advanced backcross population (BC_4_F_2_) derived from a cross of the recurrent parent 93–11 and the donor parent PA64s (Table S3). In 2015, 93–11 and CSSL*-qGMN7.1* were grown in the paddy field of Hangzhou and Hainan. Both lines were also grown in pots inside a net enclosure in Hangzhou. Each pot was filled with 4 kg of sterilized paddy soil and amended with 2 mg/kg^−1^ CdCl_2_. The soil was maintained in a flooded state before heading, then kept moist until maturity.

In the paddy field of Hangzhou (2016), flag leaf blades from 93–11 and CSSL*-qGMN7.1* were sampled at 0, 3, 6, 9, 12, 18, 24, 30, and 36 days after heading, and the grains were reaped at 6, 9, 12, 18, 24, 30, and 36 days after heading. At maturity, the aboveground parts of 93–11 and CSSL*-qGMN7.1* were reaped and segregated as grain, lemma, panicle branches, flag leaf blade, flag leaf sheath, lower leaf blade, lower leaf sheath, node, and basal stem. In the pot experiment, the flag leaf blades were harvested at heading, filling, and maturity stages, while the grains were labeled and retained to maturity. All samples were dried at 65 °C for 3 d and then weighed before determination of Mn concentration.

### Statistical analysis and QTL mapping

Statistical analysis was conducted by SAS (version 9.0). Broad-sense heritability was estimated as described by Singh and Chaudhary^38^. QTL analysis was performed with the MultiQTL package (www.mutiqtl.com) using the maximum likelihood interval mapping approach for the RILs. For major effect QTLs, the LOD threshold was obtained based on a permutation test (1,000 permutations, *P* = 0.05) for each dataset. We followed the suggestions by McCouch for the QTL nomenclature^39^.

### Fine mapping of *qGMN7.1*

To fine map *qGMN7.1*, CSSL-*qGMN7.1* was crossed with 93–11. A total of 4,943 segregating F_2_ individuals were grown in Hainan in 2015. Twelve recombinants were genotyped with previously developed insertion/deletion (InDel) markers supplied in Table S3. The progeny of these recombinants were grown and genotyped in Hangzhou (2016). Mature seeds were collected and prepared for mineral determination as described below.

### Short-term Mn uptake experiment

To compare the transport activity for Mn between 93–11 and CSSL*-qGMN7.1*, we performed a short-term (30 min) uptake experiment according to a previous method^12^. The seedlings (28 d old) of these two lines were exposed to the nutrient solution without Mn for 1 week and then subjected to the uptake solution containing various concentrations of Mn (0.0, 0.1, 0.5, 5, 10, 50, or 100 mM) at 25 °C and 4 °C with four replicates per treatment. After 30 min, the roots were washed three times with 5 mM CaCl_2_ and separated from the shoots. The roots were dried at 70 °C for 3 d and used for mineral determination as described below.

### Determination of metals in plant tissues

The dried samples were digested with a mixture of HNO_3_ (85%) and HClO_4_ (15%) at a gradient temperature (60 °C for 1 h, 120 °C for 1 h, 150 °C for 1 h, and up to 190 °C). The concentration of the metals in the digest solution was determined by atomic absorption spectrometry (Z-2000; Hitachi) and an inductively coupled plasma-mass spectrometer (7700X, Agilent Technologies) after dilution.

### Quantitative reverse transcription PCR (qRT-PCR) analysis

Seedlings of 93–11 and CSSL*-qGMN7.1* were grown in 1/2 Kimura B solution^12^ for 2 weeks, then the roots and shoots were harvested separately. *OsNRAMP5* expression was investigated in different tissues from plants grown in the paddy field at booting stage, including root, basal stem, node, lower leaf sheath, lower leaf blade, flag leaf blade, flag leaf sheath, and panicle. Flag leaves of rice varieties were sampled at the heading stage. RNA was extracted by the Micro RNA Extraction kit (Axygen) and reverse transcribed into cDNA using a ReverTra Ace qPCR-RT kit (TOYOBA, Japan). Primers for qRT-PCR were described in a previous study^12^ (Table S3). Quantitative PCR was conducted on an ABI PRISM 7900HT Sequence Detector (Applied Biosystems) according to the manufacturer’s instructions. The relative expression of each transcript was obtained by comparison with the expression of rice *actin1* (Table S3).

### Relative promoter activity assays

The promoter fragments (2 kb) of *OsNRAMP5* were amplified by PCR from the 93–11 and PA64s lines using the forward primer 5′-accatgattacgccaagcttGCGCATGTATCATTTGTTGT-3′ and the reverse primer 5′-aacgacggccagtgaattcCTCACTGCTCTCTCTCTCAA-3′, and were then cloned into the pCambia1391Z vector. The constructed pCAMBIA1391Z::93–11^p^ and pCAMBIA1391Z::PA64s^p^ plasmids were co-transformed with eGFP into rice protoplasts and transiently expressed^40^. After 12 h of incubation at 25 °C, protoplasts were collected for RNA extraction. The *GUS* expression level was detected by qRT-PCR with eGFP expression as the endogenous control.

The 35 S promoter of pAN580GFP was substituted with the *OsNRAMP5* promoters from 93–11 and PA64s. The *OsNRAMP5* promoter fragments were amplified by the forward primer 5′-aacgacggccagtgccGCGCATGTATCATTTGTTGT-3′ and the reverse primer 5′-tctagaggatccccgggtaccCTCACTGCTCTCTCTCTCAA-3′. The Pro93–11::GFP and ProPA64s::GFP plasmids were transformed into rice protoplasts and transiently expressed. After 12–16 h of incubation at 25 °C, green fluorescent signals were observed with an OLYMPUS IX71 confocal microscope.

### Plasmid construction and rice transformation

The cDNA of *OsNRAMP5* was amplified by PCR with the forward primer 5′-AAGGTACCATGGAGATTGAGAGAGAGAGC-3′ and the reverse primer 5′-AATCTAGACTACCTTGGGAGCGGGATGTC-3′, which include the *Kpn*I and *Xba*I restriction sites, respectively. The amplified fragment was cloned into the pCAMBIA1300S vector for overexpression. The constructed vector was sequenced and introduced into 93–11 by *Agrobacterium tumefaciens-* (EHA105) mediated transformation. Thirteen independent transgenic plants of pCAMBIA1300S::*OsNRAMP5* in the 93–11 background were obtained. Seedlings of these transgenic plants (T_2_ selected by hygromycin) and 93–11 were grown in 1/2 Kimura solution and transferred to pots at the four-leaf stage.

### Data availability

The datasets generated during and/or analysed during the current study are available from the corresponding author on reasonable request.

## Electronic supplementary material

Table S1-S5; Figure S1-S4
