# Supplementary material for: Characterization of a major QTL for manganese accumulation in rice grain
Source: Sci Rep. 2017 Dec 18;7:17704. doi: 10.1038/s41598-017-18090-7 (PMC5735179; doi:10.1038/s41598-017-18090-7)
Supplement: Supplementary file 1 — Table S1-S5; Figure S1-S4 [file 41598_2017_18090_MOESM1_ESM.pdf]

Title

Characterization of a major QTL for manganese accumulation in rice grain

Authors

Chaolei Liu, Guang Chen, Yuanyuan Li, Youlin Peng, Anpeng Zhang, Kai Hong, Hongzhen Jiang, Banpu Ruan, Bin Zhang, Shenglong Yang, Zhenyu Gao* & Qian Qian*

Table S1 Phenotypic variation of grain manganese (Mn) concentration in parents and RILs grown in Hangzhou and Hainan (2013).

Trait (mg/kg)		Parents		RIL population		H^2%
		93-11 (Mean ± SD)	PA64s (Mean ± SD)	Mean ± SD	Range	
Mn	Hangzhou	43.20 ± 2.24	60.42 ± 3.34**	46.88 ± 13.11	70.66	30.67
	Hainan	24.29 ± 1.94	32.18 ± 1.92**	28.37 ± 4.90	21.75	

Note: ** indicates a 1% significant level compared to 93-11 according to the *t* test (n = 6). Broad-sense heritability (H^2) was calculated as described in Materials and Methods.

Table S2 Quantitative trait loci (QTLs) for grain Mn concentration in RIL population.

QTL[a]	Chr.	Marker interval	Hangzhou			Hainan		
			LOD[b]	R^2(%)	Add.[c]	LOD[b]	R^2(%)	Add.[c]
qGMN1	1	SNP1-238~1-247	2.57	5.8	1.189			
qGMN2	2	SNP2-329~2-367	2.86	10.2	1.594			
qGMN3	3	SNP3-31~3-32				4.13	3.4	-2.414
qGMN4	4	SNP4-220~4-243	2.78	3.1	-0.385	2.52	3.5	-1.564
qGMN5	5	SNP5-211~5-225	3.92	4.8	1.115			
qGMN6.1	6	SNP6-31~6-32	2.98	3.1	-0.170			
qGMN6.2	6	SNP6-149~6-180	2.68	9.4	-1.756	3.95	13.5	-5.708
qGMN7.1	7	SNP7-53~7-64	5.85	15.6	-1.547	6.37	22.8	-6.624
qGMN7.2	7	SNP7-152~7-199	2.67	6.0	-1.204	2.82	7.3	-5.320
qGMN8.1	8	SNP8-29~8-63	4.49	10.6	-1.576	3.16	7.9	-4.662
qGMN8.2	8	SNP8-103~8-116				3.92	4.8	-2.947
qGMN9	9	SNP9-9~9-28	2.93	6.6	1.239			

Note: [a] Detected QTLs are shown with the italic abbreviation of the trait and the chromosome number. [b] Threshold values of logarithm of odds (LOD)>2.50 are shown. [c] Additive effect (Add.) of the 93-11 allele.

Table S3 Primers for Insertion/deletion (InDel) markers and qRT-PCR in this study.

Marker name	Primer sequence (5'→3') Forward	Reverse
L6952	GGGAGCCAAAACAGTAGCAG	GCTGTGCCCACTAAGTTAGC
L8286	AACTCATGTGCAAGAAGGAG	TGTTTATGCTATTATTAAGCAACCT
L8397	GCCTAAAGTATAAGGCAAATTAGA	AAAGAGATGATGGTGGGAGT
L8451	TCCCATAATCTAGACAAGGG	TAACATCACACGACAGTAC
L8472	GGTCCATCTTATTCAGTTGG	TACTCCGTATGCGTGGAACC
L8691	ACTAAGCATCACTTGTAGAG	TGTTTCAAGCTAAATGCCAC
L8728	TCCCATAATCTAGACAAGGG	TAACATCACACGACAGTAC
L8798	TCCGATGTTACAACCAAGAC	TCCTCATCTCGGTTGCTCAG
L8857	TCCTCATCTCGGTTGCTCAG	CTTTTCGCTAATTTGAACTTGT
L8906	CAAAATTTTCATGGCTTGAC	TGTAGTCTTTGATGGAAAGCTC
L8918	CAATGTATATTTGACATAAACCAACA	CTTCCTTGCCCTGGAAGC
L9020	TGAGATGAGTGAAAGGGGATA	TGAGTGATAAAACAACTGATAACAA
L9205	GGAGCTTGTGGAGTATTACAT	GGGCTCTTTGACATCGTC
L9312	AGGGTCCTCGTCTTCTTTTGA	AGTCCTATCCAGATTGCCCTG
RT-*OsNRAMP5*	CAGCAGCAGTAAGAGCAAGATG	GTGCTCAGGAAGTACATGTTGAT
RT-*Actin1*	GACTCTGGTGATGGTGTCAGC	GGCTGGAAGAGGACCTCAGG
RT-*GFP*	CCTACGGCAAGCTGACCCTG	TGCTGCTTCATGTGGTCGGG
RT-*GUS*	TCCTTCATTTTCTTGGTTAGGACCC	CGTCGGTTCTGTAACTATCATCATCA

Table S4 The code, name, subspecies and haplotypes of *OsNRAMP5* promoter of rice varieties used in the present study.

Code	Cultivar name	Subspecies	OsNRAMP5 promoter haplotype
PA64s	PA64s	*Indica*	I
NPB	Nipponbare	*Japonica*	I
WYJ7	Wuyunjing7	*Japonica*	I
CCG	Changchungu	*Indica*	I
154	OPALE	*Temperate Japonica*	I
252	Qiuguang	*Temperate Japonica*	I
276	Qiutianxiaoding	*Temperate Japonica*	I
563	SAGC-7	*Admix*	I
736	GANIGI	*Tropical Japonica*	I
992	CNA-7BO11>33-13-6-1	*Tropical Japonica*	I
993	IR 63380-16	*Tropical Japonica*	I
995	RIZZOTTO 51-1	*Temperate Japonica*	I
1057	T757	*Indica*	I
1199	BOCAO	*Tropical Japonica*	I
93-11	93-11	*Indica*	II
71	MELEKE	*Indica*	II
140	IR 72967-12-2-3	*Tropical Japonica*	II
166	WAS 170-B-B-1-1	*Indica*	II
167	WAS 173-B-B-6-2-2	*Indica*	II
271	K24	*Indica*	II
447	Chorofa	*Indica*	II
491	Zhongyouzao81	*Indica*	II
493	IR77289-14-1-2	*Indica*	II
498	IR62266-42-6-2	*Admix*	II
502	829	*Admix*	II
767	NSIC RC9 (APO)	*Indica*	II
871	ORIONE	*Temperate Japonica*	II
875	UPL RI-7	*Admix*	II
1105	Padi Ladang Ase Polo Komek	*Indica*	II
TN1	Taizhong1	*Indica*	III
NJ6	Nanjing6	*Indica*	III
565	KCD1	*Indica*	III

Note: Most of the varieties are from 3K rice germplasm resources (http://cgm.sjtu.edu.cn/3kricedb/).

Table S5 Comparison of eleven traits between 93-11 and CSSL-*qGMN7.1* in Hangzhou (2015).

Trait	93-11	CSSL-*qGMN7.1*
Plant height (cm)	120.1 ± 2.6	121.6 ± 2.1
Panicle length (cm)	24.8 ± 1.9	25.6 ± 1.5
Primary branch number	10.8 ± 0.7	10.7 ± 0.9
1000-seed weight (g)	30.02 ± 1.61	29.86 ± 1.41
Yield per plant (g)	35.9 ± 2.7	34.6 ± 1.8
Spikelets per panicle	220.3 ± 27.9	216.3 ± 18.5
Seed set	89.6 ± 0.8	90.2 ± 1.2
Productive tiller	8.3 ± 1.7	8.5 ± 2.1
Heading date(d)	92 ± 2.6	91 ± 2.3
Grain Mn conc. (mg/kg DW)	32.26 ± 2.37	43.56 ± 3.79**
Grain Cd conc. (μg/kg DW)	204.24 ± 40.26	115.24 ± 12.06**

Phenotypes are presented as mean ± standard deviation. ** indicates a 1% significant level compared to 93-11 according to the *t* test (*n* = 6).

Fig. S1

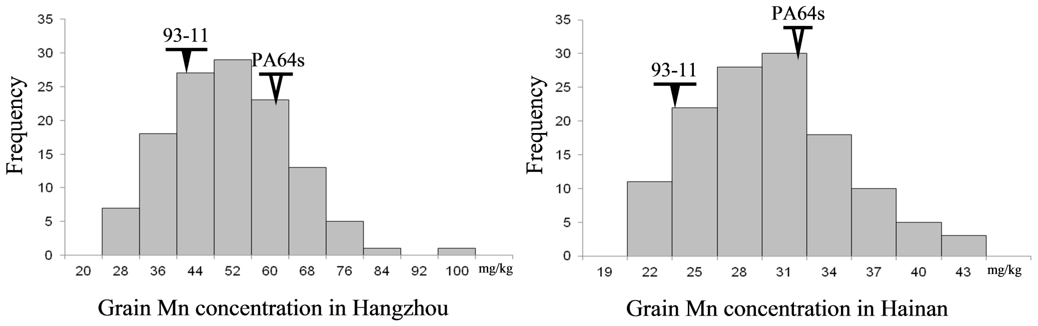

Fig.S1 Frequency distributions for grain Mn concentration in RILs in Hangzhou (2013) and Hainan (2013). Arrowheads indicate the means of 93-11 and PA64s and horizontal bars represent standard deviation of each cultivar ($n = 6$).

Fig.S2

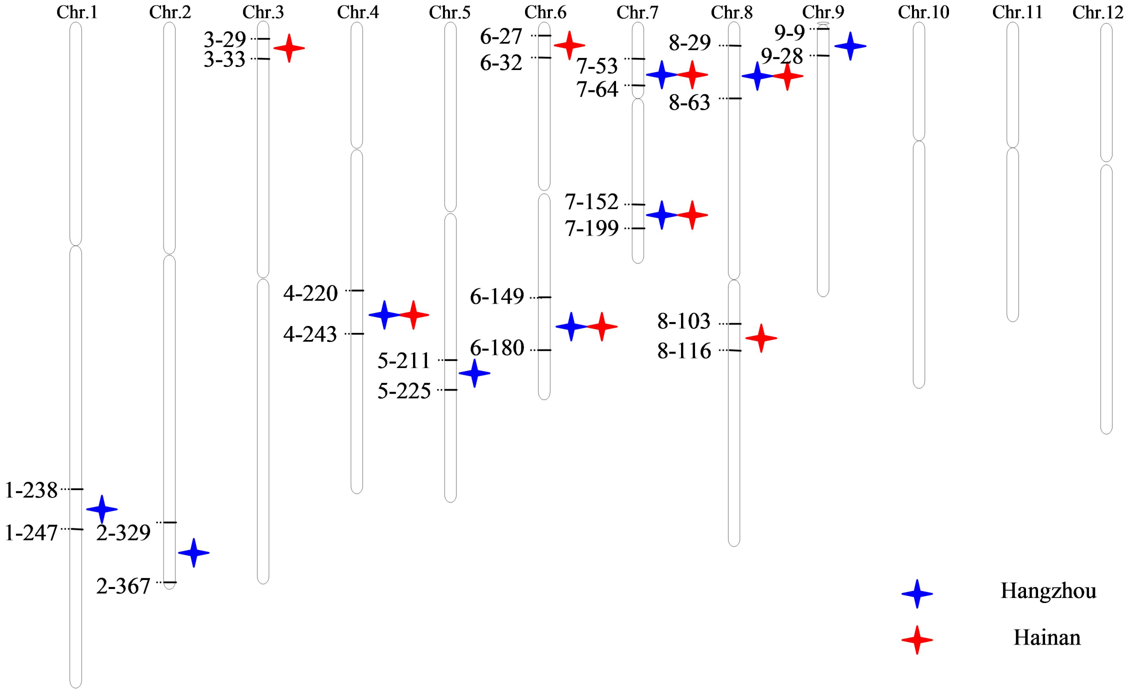

Fig.S2 Chromosomal locations of QTLs for grain Mn concentration detected in RILs. The number on the left of each chromosome is the SNP marker interval. The blue star represents phenotype collected in Hangzhou (2013) and the red one that in Hainan (2013).

Fig.S3

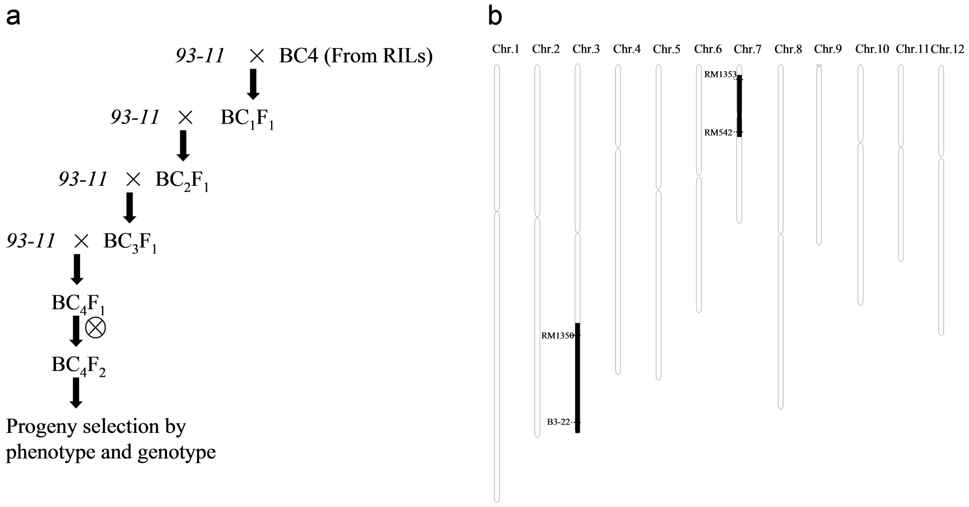

Fig.S3 Breeding scheme for the development of CSSL-*qGMN7.1* and its genetic background. (a) BC4, a line carrying segments of PA64s at *qGMN7.1* locus from RILs, was continuously backcrossed with 93-11. The CSSL-*qGMN7.1* was selected from BC_4F_2 population by phenotyping and genotyping; (b) The genetic background of CSSL-*qGMN7.1*. Black bars represented the subsitution segments from PA64s.

Fig.S4

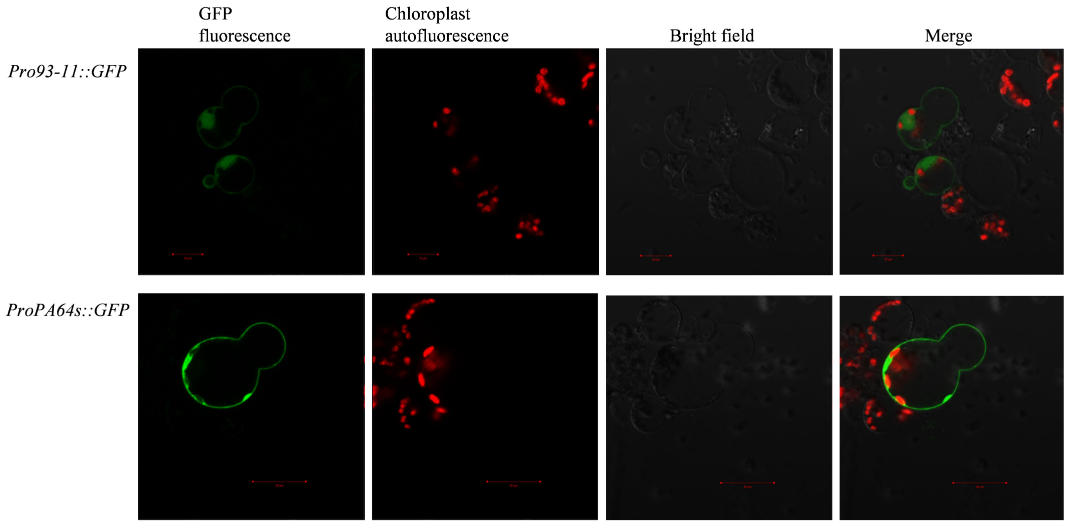

Fig.S4 Green fluorescent signals of GFP driven by 93-11 (up) and PA64s (down) promoter.
